# Follow-Up of Post-Discharge Growth and Mortality after Treatment for Severe Acute Malnutrition (FuSAM Study): A Prospective Cohort Study

**DOI:** 10.1371/journal.pone.0096030

**Published:** 2014-06-03

**Authors:** Marko Kerac, James Bunn, George Chagaluka, Paluku Bahwere, Andrew Tomkins, Steve Collins, Andrew Seal

**Affiliations:** 1 Valid International, Oxford, United Kingdom; 2 Department of Paediatrics and Child Health, College of Medicine, Blantyre, Malawi; 3 Institute for Global Health, University College London, London, United Kingdom; 4 Leonard Cheshire Disability and Inclusive Development Centre, University College London, London, United Kingdom; 5 Liverpool School of Tropical Medicine, Liverpool, United Kingdom; 6 Centre of Research in Epidemiology, Biostatistics and Clinical Research, Free University of Brussels, Brussels, Belgium; Kenya Medical Research Institute (KEMRI), Kenya

## Abstract

**Background:**

Management of Severe Acute Malnutrition (SAM) plays a vital role in achieving global child survival targets. Effective treatment programmes are available but little is known about longer term outcomes following programme discharge.

**Methods:**

From July 2006 to March 2007, 1024 children (median age 21.5 months, IQR 15–32) contributed 1187 admission episodes to an inpatient-based SAM treatment centre in Blantyre, Malawi. Long term outcomes, were determined in a longitudinal cohort study, a year or more after initial programme discharge. We found information on 88%(899/1024).

**Results:**

In total, 42%(427/1024) children died during or after treatment. 25%(105/427) of deaths occurred after normal programme discharge, >90 days after admission. Mortality was greatest among HIV seropositive children: 62%(274/445). Other risk factors included age <12 months; severity of malnutrition at admission; and disability. In survivors, weight-for-height and weight-for-age improved but height-for-age remained low, mean −2.97 z-scores (SD 1.3).

**Conclusions:**

Although SAM mortality in this setting was unacceptably high, our findings offer important lessons for future programming, policy and research. First is the need for improved programme evaluation: most routine reporting systems would have missed late deaths and underestimated total mortality due to SAM. Second, a more holistic view of SAM is needed: while treatment will always focus on nutritional interventions, it is vital to also identify and manage underlying clinical conditions such as HIV and disability. Finally early identification and treatment of SAM should be emphasised: our results suggest that this could improve longer term as well as short term outcomes. As international policy and programming becomes increasingly focused on stunting and post-malnutrition chronic disease outcomes, SAM should not be forgotten. Proactive prevention and treatment services are essential, not only to reduce mortality in the short term but also because they have potential to impact on longer term morbidity, growth and development of survivors.

## Background

Severe acute malnutrition (SAM) is a major global public health problem [1],[2]. It includes two forms of malnutrition, oedematous malnutrition (kwashiorkor) and severe wasting (low weight-for-height and/or low mid upper arm circumference) [3]. Severe wasting alone affects 18.7 million children (95% CI 14.2 to 23.2 million) aged under 5 years and is responsible for over half a million deaths per year (7.4 to 7.8% of total under-5 child deaths) [4]. Treating SAM thus has an important role to play towards global health initiatives such as Millennium Development Goal 4, decreasing child mortality [5]. For treatment services to improve their effectiveness and impact, it is vital that they are informed by high quality evidence.

Whilst there are no universal performance targets for SAM treatment programmes, there are widely acknowledged minimum standards such as those set by the ‘SPHERE’ humanitarian charter [6]. The latest version of SPHERE released in 2011 suggests that *overall* therapeutic feeding programme mortality should not exceed 10%, less than 15% should default, and over 75% of discharges should have recovered - defined as “free from medical complications; regained appetite; achieved and maintained appropriate weight gain without nutrition-related oedema, (e.g. for two consecutive weighings)” [6]. Such outcomes are certainly achievable, especially in well run ‘Community Management of Acute Malnutrition’ (CMAM) programmes which emphasize high population coverage, proactive case-finding, and early treatment [7]. (SPHERE emphasises that the interpretation of outcomes should take into account programme type: programmes which implement outpatient care alone should expect better outcomes than those which include inpatient care, the latter usually having a sicker and more vulnerable patient group). However, even if the SPHERE targets are achieved, it is important for managers, policy makers and funders to appreciate that these are short term outcomes, after a median of some 40 days of treatment. They do not necessarily reflect subsequent child survival and morbidity outcomes. The true public health impact of an intervention is also determined by how the discharged children fare in the longer term.

There is limited data on long term outcomes following discharge from SAM treatment. The few studies that have looked at this are mostly old, pre HIV and even pre current definitions of SAM [8]. This represents an important knowledge gap. Our primary aim in this study was to follow up patients over a year after their treatment for SAM to describe the longer term mortality and growth outcomes. A secondary aim was to identify key risk factors for good/poor long term outcomes, particularly those which might be amenable to change or which would help identify high risk individuals for more targeted future support. By doing this, our goal is to inform improvements in future treatment services.

## Methods

### Ethics Statement

This follow-up study was approved by the Malawi College of Medicine Research and Ethics Committee (Reference P.03/04/236). Home visits followed the example of a previous study based on MOYO [9] and had an added advantage of potential clinical benefit i.e. if any problems were identified, the carer was advised and where necessary referred.

### Study Design

This was a prospective cohort study, arising from an RCT (ISRCTN 19364765) described in detail elsewhere [10]. Briefly, the ‘ProNut study’ had enrolled malnourished children in a trial examining the efficacy of a prebiotic/probiotic mixture (Synbiotic 2000Forte™) added to ready-to-use therapeutic food during SAM treatment. No significant effects were found.

### Study setting

MOYO nutrition ward, Queen Elizabeth Hospital, Malawi. This serves a catchment population of both urban and rural Blantyre district. At the time there were no community programmes for SAM active in the district, so: no community mobilization; no proactive identification of SAM cases; no sub-classification into ‘complicated’ and ‘uncomplicated’ SAM [11]. Children diagnosed with SAM (either at local health centres or in the paediatric admissions area of Queen's hospital) were all referred for inpatient care.

Anti-retroviral (ARV) treatment for HIV had been available in Queen's Hospital since 2004, and the process of scaling up and decentralizing was happening at the time of this study. Whilst ARVs were always available, guidance at the time was for children newly diagnosed with HIV to start ARVs if meeting stage 3 or 4 (WHO classification) which included failure to respond to nutritional therapy [12]. As a result many would not have been considered for ARVs until 1–2 months after presenting with SAM, and those with good nutritional recovery not be eligible unless another staging criteria met. From October 2006 we were able to obtain CD4 counts which also informed the decision to start ARVs. Co-trimoxazole prophylaxis was provided from admission, and advised to be continued indefinitely.

### Participants

Cohort subjects were children admitted to MOYO nutrition ward for inpatient care from the 12^th^ July 2006 to 9^th^ March 2007. This was the ProNut recruitment period and included both dry (July to November) and rainy seasons (December to March). Whilst not every carer consented to their child's enrolment into ProNUT, almost all answered a detailed baseline questionnaire. Admission criteria to MOYO followed Malawi 2007 national guidelines [13]. These defined SAM as weight-for-height <70% median (NCHS growth references) *and/or* mid upper arm circumference <110 mm *and/or* oedematous malnutrition. Because of the inpatient-only set-up with children self-presenting to care, it is likely that most would have been towards the ‘complicated’ end of the SAM spectrum [11]. However, since our patient assessment at the time did not include an appetite test, an unknown (but likely small) proportion would have had ‘uncomplicated’ SAM - which in today's treatment programmes would be treated at home [2].

Children who were successfully cured (defined as two consecutive visits at above 80% weight-for-height, no oedema, clinically stable) in MOYO's combined inpatient/outpatient programme were asked to return for a ward-based review on the 1 year anniversary of their cure date. Non-cures and those failing to return for assessment were followed-up at their home by a mobile team comprising of a study nurse and driver. They used a verbal address obtained from carers during the admission. The mobile team were usually able to determine outcome information at first field visit, and only rarely needed to return if nobody was available to report on what had happened to a child.

### Variables

The main study outcome was to describe longer term post-treatment survival. This was defined as the child being seen or reliably reported to be alive at ≥1year after the original discharge date from MOYO. Deaths were subdivided into short term (whilst still an inpatient on MOYO); medium term (within the 1^st^ 90 days of admission but after inpatient care); longer term (defined as death >90 days following programme admission). Ninety days was chosen to be consistent with the upper limits of time normally spent in therapeutic feeding; 3 weeks of inpatient care and 10 weeks in the ‘outpatient therapeutic programme’ (OTP).

We also assessed long term growth as a secondary outcome. Weight-for-height, weight-for-age, and height-for-age z-scores were all assessed and compared with sibling controls. Sibling controls were identified at home visit and were defined as any child born to the same mother and living in the same household. We measured all siblings present at the time of follow-up visit but due to limited resources were not able to return for a second visit if any live siblings were away from the household at the original visit.

Additional variables were assessed at original admission to MOYO and are available with complete data collection sheets http://discovery.ucl.ac.uk/1306755/1/1306755.pdf [14]. They included clinical history; clinical signs assessed by a doctor or study clinical officer; family and socioeconomic status; HIV status (positive/negative/unknown); HIV clinical staging according to WHO criteria [12].

### Data Sources/measurement

During the initial ward admission, weight was measured using on Tanita 1582 digital scales, accurate to 20 g and checked daily. Length was measured to the nearest 1 mm on locally made boards. Baseline measurements were recorded by two independent observers, according to research standard protocols [15]. At follow-up, the same scales were used but with a portable Leicester height board, suitable for field use.

HIV testing was done on an opt-out basis as a routine part of ward protocols. It involved two ELISA rapid tests (Determine HIV-1/2 [Abbott Laboratories, USA] and Uni-Gold HIV [Trinity Biotech PLC, Ireland]), with a third (Hema Strip HIV 1/2 [Chembio Diagnostic System Inc, USA] or SD-Bioline HIV 1/2 [Standard Diagnostics Inc, Korea]) in case of discordant results. PCR for definitive diagnosis in children younger than 18 months of age was unavailable.

### Study Size

The RCT in which the cohort was nested determined the sample size [10]. To give an idea of the effect size that could be confidently detected, the original ProNut sample size of 400 per group was large enough, at 95% significance, 80% power, and accommodating 100 losses to follow-up, to detect an inter-group difference of 65% vs. 75% cure. With 1024 patients analysed in FUSAM (more than in ProNUT since not all consented for randomization and some died before the ProNut intervention began) sample size would have been sufficient for most clinically important exposures.

### Statistical methods

Data were entered in EpiData 3.1 (EpiData Association, Odense, Denmark, 2003–4). Check files helped ensure high quality data entry, e.g. by checking that variables were in-range and consistent with related variables. Key data (anthropometry, dates, final outcomes, HIV status) were double entered. Unusual results, such as a long time to final outcome, were cross checked against original patient files.

Main analyses were performed using Stata Intercooled 10.0TM (StataCorp LP, USA). Kaplan-Meier time-to-death curves are presented as ‘step-up’ curves in accordance with suggested best practice [16]. Multivariable analysis was done using Cox regression. Because survival curves for HIV seropositive and seronegative children violated the proportional hazards assumption (confirmed by a formal test using Schoenfeld residuals), complex multivariable analyses in our final results table are presented stratified by HIV. Other key variables (admission oedema, admission age, admission MUAC, admission WAZ) did not violate the assumption. The ‘*exactp*’ option within Stata's ‘*stcox*’ command was specified in all reported regression models.

Chi-square tables and approximate confidence limits for relative risk were used to examine categorical data using StatCalc (CDC, Atlanta, USA, 1993). Where cell numbers are small, Fisher exact results are noted.

### Quantitative variables

Z-scores were calculated from weight, height/length, age and sex variables using ENA for SMART software, version October 2007 [17]. Lower (more negative) z-score imply more severe malnutrition. Since children were admitted on the basis of NCHS references, we felt it appropriate to analyse using NCHS references. Given our research-standard measurement protocols using two independent observers, baseline extreme Z-scores were assumed to be real and not excluded [15]. However, with greater potential for error associated with field-based work at the final FuSAM visits, extreme final z-scores were considered more likely to be measurement errors than a child who is truly very small or very large. Following standard criteria [17], individuals with extreme z-scores on follow-up were thus excluded from anthropometry-related analysis: weight-for-height z-scores, WHZ (NCHS) <−4 or >+6 or weight-for-age z-score, WAZ (NCHS) <−6 or >+6 or height-for-age z-score HAZ (NCHS) <−6 or >+6.

For survival analysis and Cox regression, time 0 was the date of admission to MOYO. Time to FuSAM follow-up for other patients was calculated in days by subtracting date of admission from date of final outcome (alive, dead or unknown/other). If patients died within hours of admission, their length of stay was counted as 0.5 days so that they would still contribute to the final model.

## Results

**Figure 1:**
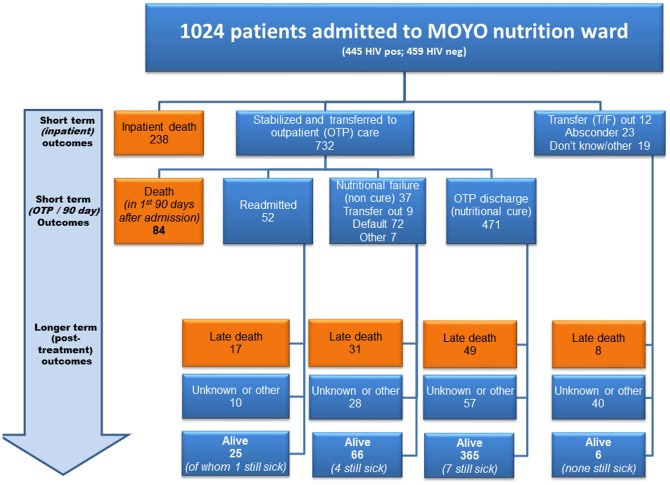
FuSAM study flow chart - all admissions to MOYO. (OTP = ‘Outpatient Treatment Programme’ – the outpatient part of treatment; T/F = transfer out to different programme). ‘Still sick’ children were seen or reported to be clinically unwell at follow-up but details were not always known.

Over the eight month study period a total of 1,024 children contributed to 1,187 malnutrition admission episodes. Final outcome information was found on 889/1024(87%). Of the 135(13%) with no 1-year outcome: 45/135(33%) could not be traced at the address given; 42(31%) did not give an address; 31(23%) had missing notes; 7(5%) lived too far away for the outreach team to visit; 10(7%) had an outcome but at less than a year. Outcomes are summarized in **figure 1**.

A total of 462/1024(45%) children were known to be still alive at a year or more after discharge from treatment. Long term survival was greatest amongst those who had been successfully cured following initial treatment. Of 471 discharged from the OTP as nutritionally cured, 365(77%) were still alive at a year or more after their first admission. Of the 427/1024(42%) who died, most did so early on in programme: 238/427(56%) of deaths were during initial inpatient treatment. Most deaths, 274/427(64%), were among children with underlying HIV infection. Differently expressed, 274/445(62%) of known seropositive children died while a total of 77/459(17%) of known seronegative children died.

**Figure 2:**
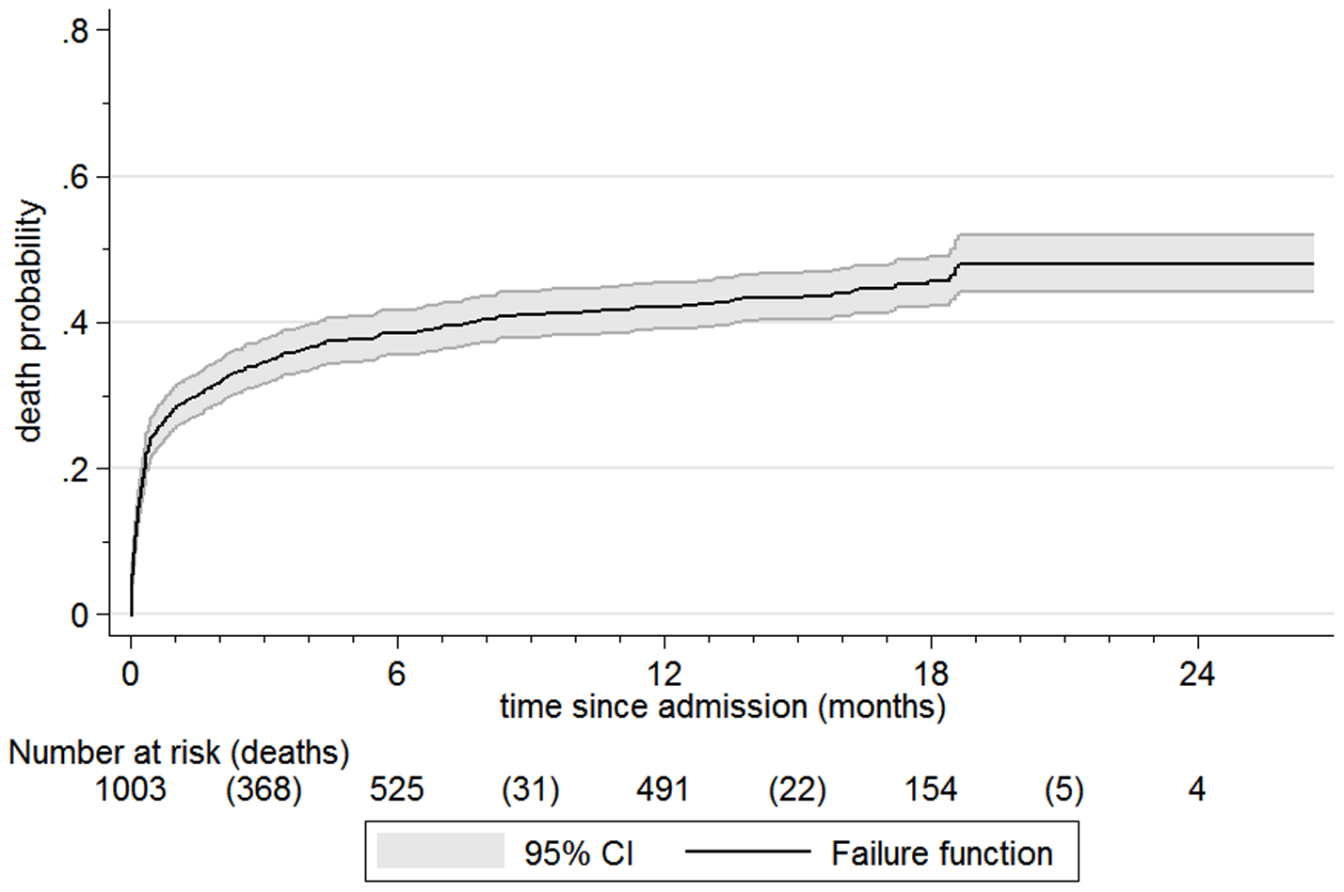
Kaplan Meier failure curve, all patients.

**Figure 3:**
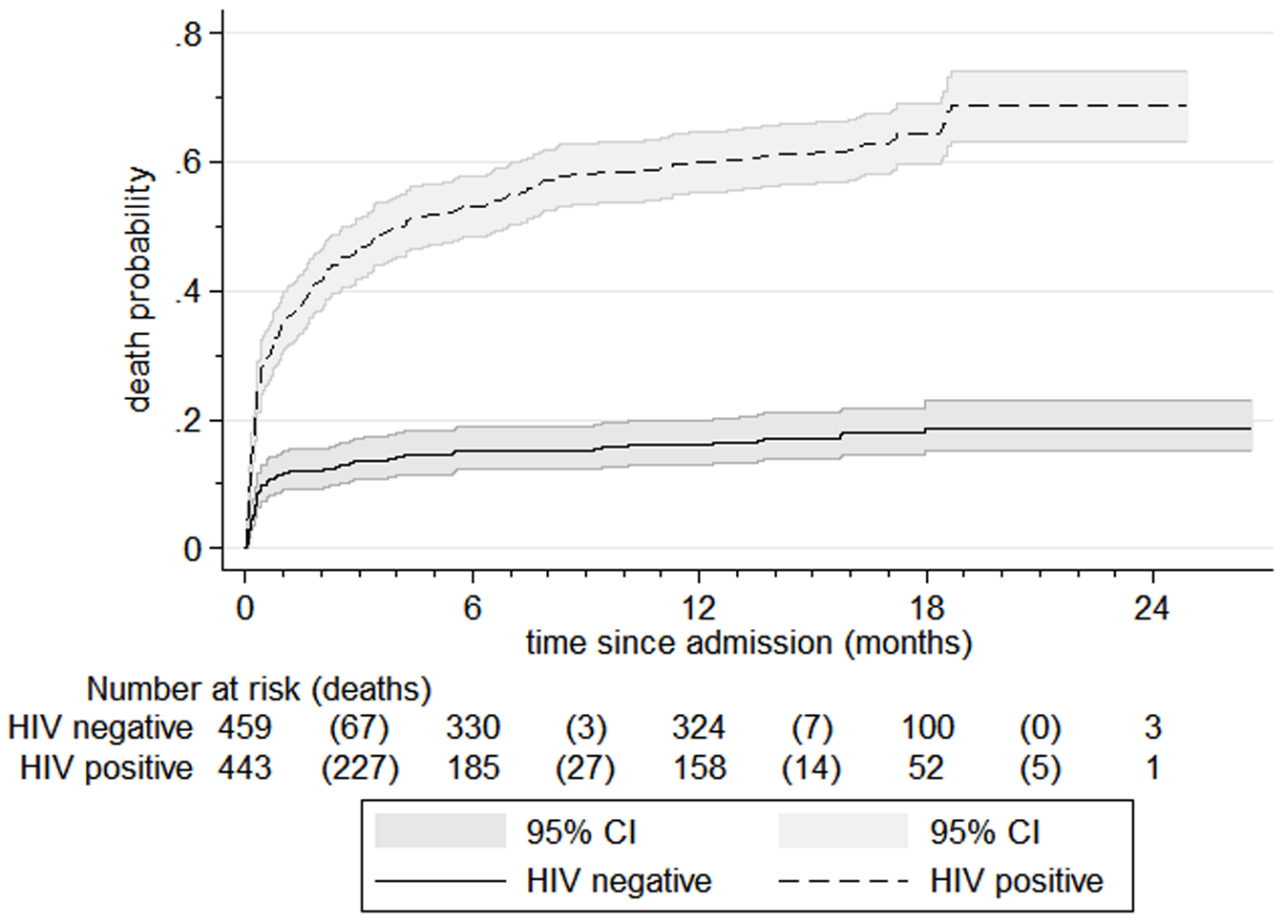
Kaplan Meier failure curves, by HIV serostatus. The tables below figures 2 and 3 show numbers at risk at the beginning of a particular time period. Deaths are in parentheses. Numbers at risk are not simply those previous at-risk minus deaths. Other outcomes also result in children being removed from further analysis (being ‘censored’). With this denominator change, the y-axis is mortality *probability* rather than percentage. Whilst our main outcomes focus is on the first year post-discharge, for completeness, data is presented until the last child's follow-up.

Kaplan-Meier failure curves in **figures 2** **and** **3** further illustrate the probability of death during different time periods. These include 1003 children: we could not plot the remaining 21/1024(2.1%) since the date of final outcome was not known. Median time from initial admission to final outcome was 287 days, IQR 11 to 529. The steep initial slope of the curve highlights the fact that most deaths happened soon after admission to programme; median time to death was 10 days, IQR 3 days to 69 days. 278/427(65%) of deaths occurred within 30 days of admission. Failure curves by HIV serostatus (**figure 3**) highlight the significant adverse impact of HIV. For both HIV negative and positive patients, most deaths occurred soon after admission. However, they soon plateau for HIV negative patients but continued to rise for those with HIV. The Log-rank test for the difference between HIV negative and positive children was p<0.0001.

**Table 1** shows the baseline profile of the cohort according to children's final outcome. Further details are available online [14]. Because admissions at the time were based on NCHS growth references, NCHS z-scores were used for subsequent calculations. To enable future researchers to compare their data against ours we also however present admission anthropometry calculated using WHO growth standards [18] (**Table S1**)

**Table 1:** Patient profile at baseline (initial admission), by final outcome.

Characteristic	Subcategory	*Death in 1^st^ 90 days (Inpatient or outpatient) (n = 322)*	*Later death (n = 105)*	*Alive at ≥1 year post admission (n = 462)*	*ALL (n = 1024)*
**Basic Demographics**	**Age in months** (median, IQR)	*19.4 (13 to 30)*	*18.8 (12 to 29)*	*23.3 (17 to 34)*	**21.5 (15 to 32)**
	**Boys**	*156/322 (48%)*	*66/105 (63%)*	*251/462 (54%)*	**543/1024(53%)**
**Type of SAM**	**Kwashiorkor** †	*175/322 (54%)*	*45/105 (43%)*	*383/462 (83%)*	**697/1024(68%)**
	**Severe wasting**	*135/322 (42%)*	*55/105 (52%)*	*69/462 (15%)*	**275/1024 (27%)**
**Anthropometry (z-scores)**	**Weight-for-height** (WHZ)	*−2.77 (1.2)*	*−2.65 (1.2)*	*−1.92 (1.2)*	***−2.25 (1.3)***
	**Weight-for-age** (WAZ)	*−4.13 (1.1)*	*−4.30 (1.1)*	*−3.18 (1.2)*	***−3.59 (1.3)***
	**Height-for-age** (HAZ)	*−3.43 (1.4)*	*−3.88 (1.3)*	*−3.03 (1.4)*	***−3.23 (1.4)***
**HIV**	**HIV seronegative**	*57 (18%)*	*20 (19%)*	*315 (68%)*	**459 (45%)**
	**HIV seropositive**	*191 (59%)*	*83 (70%)*	*139 (30%)*	**445 (43%)**
	**HIV unknown**	*74 (23%)*	*2 (2%)*	*8 (2%)*	**120 (12%)**
**Clinical**	**Disability** (any)	*24/282 (9%)*	*9/102 (9%)*	**22/453 (5%)**	**60/938 (6%)**
	**Outpatient episodes** *(last 6 months)*	*233/253 (92%)*	*90/98 (92%)*	*396/440 (90%)*	**797/884 (90%)**
	**Outpatient episodes** *(last 6 months, symptoms suggestive of malnutrition)*	*60/254 (24%)*	*26/97 (27%)*	*84/437 (19%)*	**187/882 (21%)**

† Numbers with kwashiorkor plus numbers with severe wasting = 972 rather than 1024: the remaining 52 patients had complicated moderate wasting. These children were treated according to exactly the same protocols as those with SAM and hence are included in the follow-up study.

Key observations are that: children who died were younger than those still alive at 1 year; kwashiorkor (oedematous malnutrition) was the dominant type of SAM but risk of death was lower than in non-oedematous SAM; children who were more malnourished (lower z-scores); at baseline were more likely to die; HIV was prevalent and HIV-associated mortality was high, especially with advanced disease (as indicated by low CD4 count); disability - mostly neurodisability such as cerebral palsy - was common and deaths among disabled children were high. Another observation was the large number of children who, according to their personal health record (health “passport”) or carer, were seen in an outpatient clinic in the six months prior to SAM admission,. In addition, many of these were seen for symptoms potentially consistent with SAM (e.g. “swelling”; “severely thin” or similar noted in health passport”).

Risk factors for mortality were further explored using multivariable Cox Regression in **Table 2**. Adjusting for age, oedema and HIV, low baseline MUAC, WHZ and WAZ are all strongly associated with death. Low HAZ is of borderline significance. Children with oedematous malnutrition were significantly less likely to die than those without. Though the youngest children below 12 months of age were significantly more likely to die, there was no clear age-related risk gradient among older children. HIV stands out as having the greatest adverse impact, an adjusted hazard ratio of 4.03(95% CI 3.08 to 5.25). Deaths among children whose HIV serostatus was unknown were especially high.

**Table 2:** Cox regression exploring main baseline predictors of death.

Risk factor		Number of deaths	Hazard ratio (95% CI)	Adjusted hazard ratio* (95% CI)	P (adj.)
**Sex**	Girls	202/469 (43%)	Ref.	Ref.	
	Boys	221/530 (42%)	0.92 (0.76 to 1.12)	0.89 (0.73 to 1.08)	0.26
**Age group (in months)**	> = 60	30/88 (34%)	1.11 (0.58 to 2.12)	1.22 (0.63 to 2.36)	0.55
	48–<60	13/42 (31%)	Ref.	Ref.	
	36–<48	25/68 (37%)	1.27 (0.65 to 2.49)	1.66 (0.84 to 3.29)	0.14
	24–<36	85/230 (37%)	1.26 (0.70 to 2.25)	1.38 (0.76 to 2.49)	0.29
	12–<24	176/430 (41%)	1.42 (0.81 to 2.49)	1.57 (0.89 to 2.78)	0.12
	<12	97/145 (67%)	2.89 (1.62 to 5.17)	2.49 (1.38 to 4.51)	0.002
**Oedema**	No	205/305 (67%)	Ref.	Ref.	
	Yes	220/694 (32%)	0.37 (0.30 to 0.44)	0.58 (0.47 to 0.72)	<0.001
**Mid Upper Arm Circumference**	Per cm unit increase	418/991 (42%)	0.70 (0.66 to 0.74)	0.80 (0.74 to 0.86)	<0.001
**Weight-for-Height**	Per 1 unit z-score increase	409/973(42%)	0.65 (0.60 to 0.71)	0.75 (0.68 to 0.83)	<0.001
**Weight-for-Age**	Per 1 unit z-score increase	425/999 (43%)	0.59 (0.54 to 0.64)	0.73 (0.66 to 0.81)	<0.001
**Height-for-age**	Per 1 unit z-score increase	417/989 (42%)	0.83 (0.78 to 0.89)	0.92 (0.86 to 0.99)	0.04
**HIV**	Neg	77/459 (17%)	ref	ref	
	Pos	273/443 (62%)	4.84 (3.75 to 6.24)	4.03 (3.08 to 5.25)	<0.001
	unknown	76/101 (75%)	17.8 (12.8 to 24.8)	16.9 (12.1 to 23.7)	<0.001

* Adjusted for age, oedema, and HIV status.

**Table 3** shows clinical and social factors which might plausibly explain mortality. What is notable here is that after adjusting for baseline anthropometry, very few factors remain as independent predictors of death. Disability stands out as strongly and significantly associated with death in HIV negative patients, and only just outside the p<0.05 threshold for HIV positive patients. Severe anaemia (PCV<10) was also associated with increased risk of death, but in HIV positive patients only.

**Table 3:** Cox regression exploring clinical and social risk factors for death, by HIV serostatus.

Risk factor (assessed at original admission)		Hazard ratio for all mortality (short and long term)*			
		HIV negative	p	HIV positive	p
***CLINICAL RISK FACTORS***					
**Symptoms in previous 2 weeks**	Fever	0.96 (0.58 to 1.58)	0.87	0.85 (0.65 to 1.12)	0.25
	Diarrhoea	1.59 (0.95 to 2.68)	0.08	1.15 (0.88 to 1.51)	0.31
	Vomiting	0.84 (0.52 to 1.37)	0.49	1.23 (0.96 to 1.58)	0.10
	Fast or difficult breathing	1.12 (0.59 to 2.12)	0.72	0.71 (0.51 to 0.99)	0.04*
	Cough	1.06 (0.65 to 1.74)	0.80	1.03 (0.79 to 1.36)	0.80
	Anorexia	0.75 (0.46 to 1.22)	0.25	1.17 (0.91 to 1.51)	0.22
	Flaky paint dermatosis	1.66 (0.93 to 2.96)	0.09	1.14 (0.75 to 1.72)	0.54
**Anaemia**	Any (PCV<30)	1.08 (0.65 to 1.80)	0.76	1.05 (0.80 to 1.36)	0.73
	Severe (PCV<10)	2.19 (0.66 to 7.23)	0.20	2.62 (1.18 to 5.84)	0.02*
**Malaria**		0.26 (0.04 to 1.88)	0.18	0.94 to 0.46 to 1.95)	0.88
**Ever had TB**		2.81 (0.35 to 22.7)	0.33	1.27 (0.77 to 2.09)	0.35
**Disability (any)**		2.77 (1.43 to 5.34)	0.002*	1.76 (0.94 to 3.28)	0.08
**Not breastfed (<2 year olds only)**		0.67 (0.36 to 1.26)	0.21	0.98 (0.70 to 1.38)	0.92
**Inpatient episodes** *(non-SAM, past year)*		1.03 (0.55 to 1.91)	0.94	1.31 (0.99 to 1.75)	0.06
**Outpatient episodes**	in past 6 months	0.69 (0.34 to 1.40)	0.30	0.69 (0.40 to 1.21)	0.20
*past 6 months, symptoms of SAM*		1.33 (0.74 to 2.41)	0.34	1.00 (0.74 to 1.36)	1.00
***SOCIAL AND ECONOMIC RISK FACTORS***					
**Orphan**	*Mother died*	0.97 (0.35 to 2.70)	0.95	0.72 (0.45 to 1.15)	0.17
	*Father died*	1.33 (0.52 to 3.42)	0.56	1.35 (0.84 to 2.16)	0.21
	*Both died*	2.78 (0.92 to 8.34)	0.07	1.07 (0.47 to 2.45)	0.17
**Maternal education**	*None*	Ref.		Ref.	
	*Primary school*	1.06 (0.53 to 2.13)	0.87	0.78 (0.51 to 1.20)	0.27
	*Secondary school*	0.57 (0.21 to 1.54)	0.27	0.97 (0.60 to 1.57)	0.91
**Paternal education**	*None*	Ref.		Ref.	
	*Primary school*	2.84 (0.38 to 21.26)	0.31	0.89 (0.39 to 2.04)	0.79
	*Secondary school*	2.31 (0.30 to 17.53)	0.42	0.80 (0.35 to 1.84)	0.60
**Wealth quintile**	*Poorest*		Ref.		Ref.
	*2^nd^ poorest*	1.35 (0.63 to 2.89)	0.43	0.67 (0.41 to 1.09)	0.10
	*Middle*	1.11 (0.50 to 2.44)	0.80	0.95 (0.61 to 1.47)	0.82
	*2^nd^ richest*	1.49 (0.69 to 3.26)	0.31	0.93 (0.59 to 1.45)	0.75
	*Richest*	0.95 (0.38 to 2.42)	0.92	0.91 (0.60 to 1.38)	0.66
**Main household water source**	*Piped*	Ref.		Ref.	
	*Borehole*	0.72 (0.42 to 1.23)	0.23	0.90 (0.67 to 1.21)	0.50
	*Well or spring*	1.09 (0.51 to 2.32)	0.83	0.90 (0.60 to 1.37)	0.63
**Rural Residence**		0.67 (0.39 to 1.13)	0.13	0.82 (0.62 to 1.10)	0.62

* Adjusted for oedema, age, sex, admission WAZ and admission MUAC.

**Figure 4:**
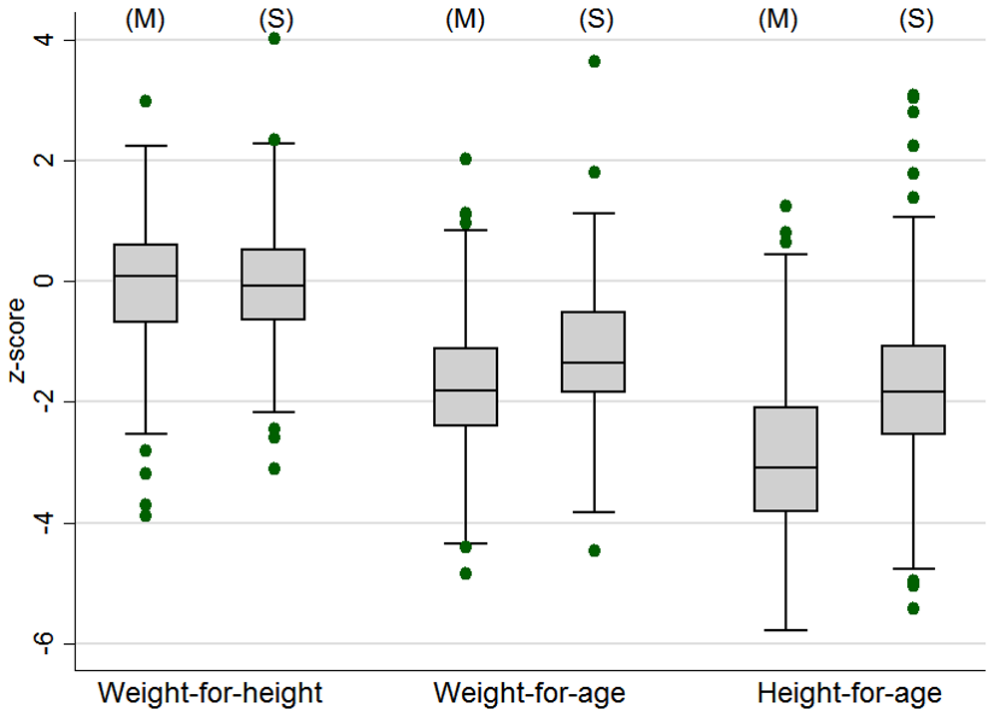
Boxplot showing weight-for-height, weight-for-age and height-for-age of the ex-malnourished surviving child (M) (n = 386) compared to sibling controls (S) (n = 277).

Finally, **Figure 4** shows the final anthropometry of the children who had received treatment on the MOYO ward, compared to sibling controls. Median birth order of the MOYO children was 2^nd^ born. 184 of the ex-MOYO children had one or more siblings measured and with valid anthropometric z-scores: 111 had only one sibling; 54 had two siblings, 18 had three siblings and one had four siblings (total 277 sibling controls). 12 sibling WHZ scores, 65 WAZ scores and 25 HAZ scores were excluded as extreme by our cleaning process. Over 90% of siblings were reported as never having had SAM themselves. Several points are of note. First is the complete catch-up of weight-for-height (WHZ). From an initial programme discharge mean WHZ of −1.96(SD 1.5), there was a 1.92(95% CI 1.76 to 2.08) z-score catch-up, bringing the surviving group to a WHZ of 0.04(1.0), comparable to sibling controls. Weight-for-age also improved by 1.66(95% CI 1.50 to 1.82) z-scores to a ‘long-term’ final value of −1.77(1.1). This was, however, significantly below that of sibling controls: mean difference in z-scores −0.55(95% CI −0.71 to −0.38, p<0.01). Height-for-age also improved, albeit by only 0.37(95% CI 0.21 to 0.53, p<0.01) z-scores, to a ‘long-term’ final value of −2.97(SD1.3). This was again significantly lower than that of the sibling controls: mean difference −1.13(95% CI −1.34 to −0.93, p<0.01). Median age of all the siblings who were measured was 61 months and median age of the ex-MOYO children who has a sibling measured was 41 months. Further details of growth outcomes are available online and include sibling-by-sibling comparisons (pages 123–125) and comparisons by HIV and admission oedema (pages 114–118) [14]. **Table S2** in this paper shows main anthropometric changes in the ex-MOYO children over the course of the study.

## Discussion

In our SAM treatment programme, overall mortality was high in both the short and medium term, markedly above SPHERE targets of <10% [6]. Long term survivors showed good weight-for-height catch-up growth, some weight-for-age catch-up but minimal height-for-age catch-up, compared to sibling controls. These observations are rare in the SAM literature and therefore offer key lessons for policy makers and programme managers:

First is the need for more information on longer term outcomes so as to assess the true impact of SAM on child mortality. In our setting, routine reporting would have given a falsely optimistic impression of post-SAM mortality. Overall 427/1024(42%) children died, but 105(25%) of these deaths occurred >90 days after their original admission. They would have been missed by most nutrition programmes which only follow-up patients up to discharge from outpatient care. Other factors which can mislead routine reporting systems but which we were able to describe include: defaulter follow-up so as to ascribe correct survival status; correctly capturing readmissions (of our 52 readmissions, 17(33%) died) – if not done, denominator population is overestimated and % mortality thus underestimated.

The second key lesson is that patient clinical profile needs to be better accounted for, both when managing individual patients and when judging programme performance. Simple targets like those set by SPHERE offer useful ‘red flags’ for further investigation but cannot alone distinguish between poorly performing programmes and those caring for a clinically complex patient population. Differentiating these will become increasingly important as countries/regions develop and primary SAM due to food insecurity alone becomes less common. A greater proportion of affected children in such areas will have an underlying medical condition either directly causing or significantly contributing to SAM. Whilst our overall mortality was high, sub-analysis by HIV status revealed that HIV infection was associated with much of the excess mortality. We note the even higher mortality among children whose serostatus was unknown. Too sick to be tested before they died, we believe that most of this group were likely to have been serostatus positive - if so, the known positive results in our paper are a likely under-estimate of the true adverse impact of HIV. Some might argue that children with HIV are therefore better described as dying from ‘end-stage HIV disease’ (AIDS) rather than from malnutrition with underlying HIV – but to us such distinctions are semantic. What matters is how to better support these children in future programmes: even with more proactive ARV treatment programmes identifying and treating children at an earlier stage, some will end up malnourished and enrolled in feeding programmes such as ours. Therefore, our results, and our message that nutrition programmes need to better care for this especially vulnerable subgroup remains valid. This HIV-associated risk is already well known [19],[20] but other factors are less well described. Notable in FuSAM is the importance of underlying disability. This affected 6% of children and accounted for significant excess mortality - a likely underestimate given that we had no formal screening tool and therefore identified only the most obvious problems. At present, few programmes actively look for disability so this is a key area for future work [21],[22].

The third message relates to the current international emphasis on tackling childhood stunting, championed by the SUN (Scaling up Nutrition) movement [23]. Our results suggest that SUN must take SAM seriously, just as SAM treatment programme should aim to engage more with the SUN. Current treatments for SAM are clearly important and necessary to minimise mortality, but may not be sufficient to address stunting on their own. Whilst it was encouraging to observe good chances of longer term survival following an initial nutritional cure (365/471(77%) of cures being still alive at a year or more after their first admission), limited improvement in survivors' height-for-age was, however, disappointing, as was their significant stunting compared to sibling controls. This is even more notable given the siblings' older median age: population prevalence of stunting increases over the first two years of life so that older cohorts might be expected to be more rather than less stunted [24]. Whilst weight-for-age had improved, it also remained low, significantly below siblings'. The marked improvement of weight-for-height z-scores though in one sense welcome, likely reflects rapid weight gain combined with poor linear growth, a pattern which may be associated with later obesity and chronic disease [25],[26]. This needs further study.

### Strengths and consistency with other literature

Our biggest strength is duration of follow-up, at least one year, often longer, after discharge from SAM treatment. Such longer term outcome information is rare. Related to this, our second major strength is a high follow-up rate – 87% is especially good for a developing country setting. This reduces the risk of bias associated with large numbers of ‘defaulters’, who may be deaths [9].

FuSAM compares favourably to the few other studies looking at longer term SAM outcomes. Most of these other studies however took place pre-HIV [8], FuSAM thus filling a key gap, regarding HIV-era outcomes, especially in sub Saharan Africa where the problem is greatest [27].

In Niger in 1992 [28], of 174 discharged from a TFP, 107(61%) were followed up. At 3–16 months follow-up 17% had died. Though compared to FUSAM this appears better, there is much larger probability of bias due to 39% unknown outcome, a proportion of whom may have been deaths. In Zaire in 1987 [29], 171 SAM children discharged from hospital were followed up and 81.6% found still alive at the end of 5 years. However, kwashiorkor in that study was mainly based on serum albumin and some of the children classified as such might not have met today's oedema, WFH, or MUAC criteria for SAM. In Tanzania in 1987, 87% of 566 children who had been discharged from an NRU were followed over a year [30]. The mortality after discharge was 8% and relapse rate was 13%. In a retrospective cohort study in Guinea Bissau, 1995 [31], 1038 severely malnourished children (defined by weight-for-age <60% NCHS standards) were followed up over 3 years. 354 had received nutritional rehabilitation whereas 684 did not, due to limited programme capacity. Up to 3 years, the relative risk of death in the rehabilitated group was 0.75(0.59 to 0.99). The mortality difference was greatest in the first three months. Weight-for-age z-scores improved from a baseline of −4.52 to −2.76. This is consistent with the weight-for-age improvements we observed in FuSAM.

### Weaknesses and generalizability

We acknowledge the limitations of our study so hope that it will inspire more detailed future work to address these:

FuSAM was conducted when CMAM (Community Management of Acute Malnutrition) was not as widespread as it is today. It was not available in Blantyre district where we worked (though it did start shortly after, in mid-2008). This public health orientated approach to SAM treats children mainly as outpatients [2], and emphasises proactive identification through community screening, rather than reactive detection during hospital visits as we had at the time of FuSAM [7]. Even though some risk factors for mortality (e.g. HIV, disability) are likely to be found in both settings, care is needed before generalising our findings to CMAM programmes. However, our results are consistent with CMAM's key principle that earlier treatment is better [7] (i.e. the less severe the malnutrition at baseline, the better the chances of survival). This has recently been confirmed in a follow-up of a CMAM cohort [8], also from Malawi showing much better outcomes than ours. Given this context, our paper is a clear call to any programmes which have not yet switched from inpatient-only SAM care to CMAM, to do so. Whilst in many ways a typical resource poor developing country setting, MOYO ward at the time of FuSAM had inputs and resources (in terms of staff time, clinical expertise, continuity of drug, food and equipment supplies) far above the vast majority of TFPs in Malawi and elsewhere in Sub-Saharan Africa. If our outcomes were poor, they are likely even worse in most other, less well supported settings.

Another limitation related to generalizability is that we examined patients already enrolled in SAM treatment. In-programme risk factors such as we observed are not necessarily the same as those at population-level. Even if a particular risk factor (e.g. HIV infection) is relevant to both general population and within-programme children, the magnitude of effect is likely to be different.

Related to the lack of obvious ‘impact’ of factors like socioeconomic status (SES), we also acknowledge that we did not model all the possible causal pathways. For instance, low SES could cause/contribute to low admission anthropometry, which did adversely affected outcomes. Hence adjusting for anthropometric status might have concealed the impact of SES. Such pathways need exploration in future work

Resource limitations meant that we were not able to follow-up regularly at fixed time-points after programme discharge, but instead relied on a single long term FuSAM visit. It is possible that some variables such as socioeconomic status or orphaning may have changed over time and hence we may have missed their effects.

Next, is the possibility of systematic reporting or recall bias. We cannot exclude this but do not believe that it is a major problem since death is a very objective outcome, with major findings such as those concerning HIV and disability all highly plausible biologically as well as themselves objective/backed by recorded blood test in case of HIV. We also cannot be sure how missing data from the 13% of children lost to follow-up might have affected results - though as previously stated this is good for a developing country setting so we do not believe it would have significantly altered our key results or affected conclusions. More of a problem is the increased data ‘noise’ inherent to some of the more subjective, self-reported variables such as socioeconomic status: small but real risks may have been obscured. Another issue is potential selection bias of children seen in the hospital admission departments, before arrival at the MOYO nutrition ward. Very sick patients might die in the admissions area or on the special care ward before transfer to MOYO. Borderline cases might be missed and wrongly sent to the normal paediatric wards instead of to MOYO.

The last potential limitation of our study relates to seasonal variations in the risk of malnutrition. Though recruitment was spread over most of a year and captured both rainy (hungry) season and dry (post-harvest) seasons, there may have been variations in nutritional status depending on when a child was admitted and followed up. Logistic constraints led to variations in time to follow-up. Had a constant time been possible, perhaps different magnitudes of z-score change might have been observed. Again, we doubt that the overall pattern of findings would have been very different. One of the advantages of using sibling controls was that they were measured at the same time as the ex-MOYO child. Where differences were seen they cannot therefore be ascribed solely to seasonal variations.

## Conclusions and implications for policy and practice

Although this is an observational study, we believe that our findings have strong policy and practice implications.

The first is the need to be much more aware of longer term as well as short term outcomes of SAM. This long term perspective will also help focus attention on important non-mortality outcomes such as growth, development, co-morbidities and pre-determinants for eventual adult non-communicable disease.

Second is that a more holistic view of SAM management is needed. Treatment is not just about food - this is necessary but not alone sufficient. Programmes should work closer in collaboration with other child health services and form part of a seamless ‘continuum of care’. Many SAM treatment programmes already appreciate the need for close links with HIV-related services [32] but the need to improve outcomes for children with disability (and likely other) chronic underlying disease is also clear from our results. Urgent future work is needed to elaborate exactly which interventions are most effective and most cost-effective for these vulnerable individuals.

Finally, our results support calls for on early identification and treatment of SAM. The pioneers of CMAM stressed community engagement and active case finding to identify and treat SAM as early as possible [33],[34]. This emphasis has not always been maintained in subsequent programmes documents [2]. Now is the time for this message to be re-vitalised in all SAM programming [7]. Whilst other confirmatory research is urgently needed, it is very plausible that earlier treatment of SAM would favourably affect stunting and longer term post-SAM chronic disease – both key areas in current international malnutrition policy and programming. Proactive SAM treatment services are essential. They not only have great potential to reduce mortality in the short term but also great potential to impact on longer term morbidity consequences.

## Supporting Information

Table S1 **Admission Anthropometry by NCHS growth references and WHO growth standards.**
(DOCX)

Table S2 **Anthropometry over the course of the study (NCHS growth references).**
(DOCX)
