# Supplementary material for: Follow-Up of Post-Discharge Growth and Mortality after Treatment for Severe Acute Malnutrition (FuSAM Study): A Prospective Cohort Study
Source: PLoS One. 2014 Jun 3;9(6):e96030. doi: 10.1371/journal.pone.0096030 (PMC4043484; doi:10.1371/journal.pone.0096030)
Supplement: Table S1 — Admission Anthropometry by NCHS growth references and WHO growth standards. (DOCX) [file pone.0096030.s001.docx]

Table S1 Admission Anthropometry by NCHS growth references and WHO growth standards

|  | NCHS growth reference  Mean (sd) | NCHS  Min max | WHO growth standard  mean (sd) | WHO  Min max |
| --- | --- | --- | --- | --- |
| **Weight-for-height** (WHZ) | **-2.25 (1.3)**  *n=976* | -5.757 1.492 | **-2.76 (1.9)**  *n=916* | -8.985 1.932 |
| **Weight-for-age** (WAZ) | **-3.59 (1.3)**  *n=1003* | -6.572 .923 | **-3.76 (1.6)**  *n=983* | -7.829 1.362 |
| **Height-for-age** (HAZ) | **-3.23 (1.4)**  *n=992* | -8.216 2.648 | **-3.50 (1.5)**  *n=992* | -8.841 3.395 |

*Please note that n is slightly different in the different groups due to missing values and also different valid ranges for NCHS and WHO growth standards
