# Supplementary material for: Follow-Up of Post-Discharge Growth and Mortality after Treatment for Severe Acute Malnutrition (FuSAM Study): A Prospective Cohort Study
Source: PLoS One. 2014 Jun 3;9(6):e96030. doi: 10.1371/journal.pone.0096030 (PMC4043484; doi:10.1371/journal.pone.0096030)
Supplement: Table S2 — Anthropometry over the course of the study (NCHS growth references). (DOCX) [file pone.0096030.s002.docx]

Table S2 Anthropometry over the course of the study (NCHS growth references)

|  | **Mean z-score (SD)** | | | **Difference**  **(95% CI)** | **P value** |
| --- | --- | --- | --- | --- | --- |
|  | **At admission** | At programme discharge | At 1 year follow-up | *1 year – programme discharge* |  |
| Weight-for-height | **-2.25 (1.3)**  n=976 | -1.96 (1.5)  n=966 | -0.04 (1.0)  n=386 | 1.92 (1.76 to 2.08) | <0.0001 |
| Weight-for-age | **-3.59 (1.3)**  n=1003 | -3.42 (1.4)  n=993 | -1.77 (1.1)  n=386 | 1.66 (1.50 to 1.82) | <0.0001 |
| Height-for-age | **-3.23 (1.4)**  n=992 | -3.34 (1.4)  n=983 | -2.97 (1.4)  n=386 | 0.37 (0.21 to 0.53) | <0.0001 |
